# Investigation of the Optimal Dose aPCC in Reversing the Effect of Factor Xa Inhibitors—An In Vitro Study

**DOI:** 10.1177/10760296211021156

**Published:** 2021-06-01

**Authors:** Nina Haagenrud Schultz, Jawed Fareed, Pål Andre Holme

**Affiliations:** 1 Department of Haematology, Oslo University Hospital, Oslo, Norway; 2 Research Institute of Internal Medicine, Oslo University Hospital, Oslo, Norway; 3 Department of Haematology, Akershus University Hospital, Lørenskog, Norway; 4 Department of Pathology, Loyola University Medical Center, Maywood, IL, USA; 5 Institute of Clinical Medicine, University of Oslo, Oslo, Norway

**Keywords:** apixaban, rivaroxaban, FXa inhibitors, activated prothrombin complex concentrate, reversal

## Abstract

Factor (F) Xa inhibitors are safe and effective alternatives to warfarin. There are concerns about the lack of a reversal strategy in case of serious bleeds or need for emergency surgery in situations when the antidote andexanet alfa is not available. Factor concentrates are widely used, but there are few clinical studies regarding the reversal effect of activated prothrombin complex concentrate (aPCC). Because of the feared thrombogenicity, administration of the lowest effective dose would be desirable. To determine the lowest concentration of aPCC sufficient to reverse the effect of rivaroxaban and apixaban. Blood from 18 healthy volunteers were supplemented with apixaban or rivaroxaban. aPCC was added to obtain 10 different concentrations ranging from 0.08-1.60 U/mL. Thromboelastometry and thrombin generation assay were used to assess the reversal effect. aPCC concentrations of 0.08 and 0.16 U/mL restored thromboelastometry clotting time to baseline in apixaban (*P* = 1.0) and rivaroxaban (*P* = 1.0)-containing samples, respectively. The concentrations 0.08 U/mL (*P* = 0.5) and 0.24 U/mL (*P* = 0.2) were sufficient to restore thrombin generation. Concentrations of 0.56 U/mL and higher, caused significantly higher ETP than baseline in apixaban-containing samples (*P* < 0.05). aPCC concentrations lower than previously reported were effective in reversing the effect of FXa inhibitors *in vitro.*

## Introduction

Direct-acting oral anticoagulants (DOACs), including the factor Xa (FXa) inhibitors rivaroxaban and apixaban, have been proven to be effective and safe alternatives to warfarin in treatment of venous thrombosis and prevention of stroke in patients with atrial fibrillation.^1,2^ These drugs exert their anticoagulant effect by binding FXa and consequently inhibiting the conversion of prothrombin to thrombin. Although it has been shown that the associated bleeding risk is as low or lower for apixaban and rivaroxaban than for warfarin, spontaneous and trauma-induced bleeding episodes under treatment of FXa inhibitors do occur.^3^ The prescription rate of FXa inhibitors has increased greatly the last few years,^4,5^ and patients on FXa inhibitors suffering from episodes of major bleeding or in need of emergency surgery are frequently encountered in emergency rooms.^6,7^

In 2018, the antidote andexanet alfa was approved in the US followed by many countries in Europe in 2019. Andexanet alfa neutralizes the anticoagulant effect of both direct FXa inhibitors and indirect FXa inhibitors like heparin and low molecular weight heparin (LMWH).^8,9^ However, this antidote has some disadvantages as it is not available in all countries and hospitals, it is costly and there is a concern about the thrombogenicity. There are also situations where andexanet alfa is unsuitable, for example in case of intolerance or preoperatively to open heart surgeries requiring cardiopulmonary bypass and therefore the need for heparin infusion.

Guidelines suggest prothrombin complex concentrate (PCC) or activated PCC (aPCC) as alternative hemostatic agents to reverse the FXa inhibitor effect.^10–13^ Observational studies have shown that both these agents are relatively effective and safe treatment options in case of life threatening bleeding or need for emergency surgery in FXa inhibitor-treated patients.^14–19^ There are no comparative studies with these drugs, but laboratory studies have demonstrated a better reversal effect achieved by aPCC than PCC.^20–22^ In addition to FII, FIX and FX, mainly in non-activated forms, aPCC contains activated FVII and traces of FXa and thrombin.^23^ The doses suggested to reverse the effect of FXa inhibitors are varying from 25 to 50 U/kg, but some studies have indicated that the lower dose may be sufficient.^20,24,25^

There has been a concern about the thrombogenicity of aPCC, but the limited clinical data available indicate that the rate of thrombosis is no higher for aPCC than other reversal strategies.^9,14,15,17^ However, patients treated with anticoagulants are already at risk of thrombotic events, and reversal of the anticoagulant effect with a hemostatic agent in the lowest effective dose would be preferable. The aim of this study was to find the concentration of aPCC sufficient to reverse the anticoagulant effect of rivaroxaban and apixaban.

## Methods

### Healthy Donors and Blood Collection

Healthy volunteers (n = 18) with ages between 18 and 85 years old and no history of vascular disease were included. Exclusion criteria were previous thromboembolic disease, an abnormal bleeding history or ongoing treatment known to affect hemostasis, for example anticoagulants, anti-platelet drug(s), non-steroidal anti-inflammatory drug(s), hormone therapy or selective serotonin reuptake inhibitors (SSRI). All the participants gave written informed consent, and the study was approved by the Norwegian regional committee for medical and health research.

Blood collection was performed with minimal stasis by a 21Gx19 mm butterfly needle (Vacuette^®^ Greiner Bio-One GmbH, Kremsmünster, Austria) at 2 different occasions. After the first blood sampling, whole blood was spiked with apixaban and after the other occasion, whole blood was spiked with rivaroxaban. Blood for thromboelastometry assessment was collected using 3 mL tubes containing 0.3 mL with 0,109 M buffered citrate (Monovette^®^, Sarstedt, Nümbrecht, Germany) prefilled with additional Corn Trypsin Inhibitor (CTI) (Hematologic Technologies Incorporates, Essex Junction, VT, USA) at a final concentration of 20 µg/mL. Blood samples for thrombin generation measurements were collected in 4.5 mL Vacutainer^®^ tubes (Becton-Dickinson, Franklin Lakes, NJ, USA) containing 0.5 mL with 0.109 M buffered citrate without CTI.

### Study Agents and Blood Processing

Apixaban (Bristol-Myers Squibb, Princeton, NJ, USA) was dissolved in demineralized water and thereafter diluted to a concentration of 110 µg/mL. Rivaroxaban (Bayer Pharma, Leverkusen, Germany) was dissolved in dimethyl sulfoxide (DMSO) as it was insoluble in water. The final DMSO concentration was 0.05% and rivaroxaban 110 µg/mL. aPCC (FEIBA®, Baxter AG, Vienna, Austria) was reconstituted with 20 mL sterile water according to instructions from the manufacturer. The concentrations of apixaban and rivaroxaban were chosen to mimic peak concentration values in FXa inhibitor-treated patients, 200 ng/mL for apixaban and 250 ng/mL for rivaroxaban.^26^ One aliquot was spared which served as “baseline.”

Activated prothrombin complex concentrate (aPCC) was supplemented to the aliquots to obtain samples with final concentrations of 0.08 to 0.8 U/mL, which correspond to doses ranging from 5 to 50 U/kg. The doses were calculated assuming that a human contains 0.65 ml blood/kg. For the rivaroxaban-containing samples, 1 aliquot with an even higher concentration of aPCC was prepared (1.60 U/mL, corresponding to a dose of 100 U/kg).

Platelet poor plasma (PPP) was obtained by double centrifugation (10 min at 2000xg in room temperature (RT) and after careful removal of the supernatant another centrifugation for 10 minutes at 10 000xg in RT). The remaining whole blood was incubated at 37°C for 30-90 min before performing the thromboelastometry measurements.

### Thrombin Generation Assay (TGA)

Thrombin generation was measured in PPP using the Calibrated Automated Thrombogram (CAT) TGA (Diagnostica Stago, Asnières-sur-Seine, France) with the Thrombinoscope software (Thrombinoscope BV, Maastricht, The Netherlands). PPP containing FXa inhibitors and aPCC in different concentrations, including 1 unspiked sample, were run in triplicates. The PPP reagent containing 5 pM tissue factor (TF) and 4 µM phospholipids (Diagnostica Stago, Asnières-sur-Seine, France), was used to initiate thrombin generation.^27,28^

### Thromboelastometry

Clotting in whole blood was initiated with minimal TF activation.^29–31^ Clotting time (CT; seconds), clot formation time (CFT; seconds) and maximum clot firmness (MCF; mm) were measured by ROTEM^®^ (TEM Innovations, Munich, Germany. The plastic test cups were prepared with 40 uL buffer, i.e. a mixture of equal parts of buffer 1 (20 mM Hepes, 150 mm NaCl, pH 7.4) and buffer 2 (20 mM Hepes, 150 mM NaCl, 200 mM CaCl_2_, pH 7.4). Recombinant relipidated TF (Innovin^®^, Dade Behring, Liederbach, Germany) diluted in a total volume of 20 uL of buffer 1 was also added. To initiate the reaction, whole blood (280 uL) was added to the plastic cup. The total volume of reagents and whole blood in each cup was 340 µL, thus the final TF dilution was 1:70 000, corresponding to a theoretical TF concentration of 0.35 pM. The measurements were run in duplicates.

### Anti-FXa Activity Measurements

The concentration of rivaroxaban and apixaban was measured by a rivaroxaban-or apixaban-calibrated anti-Xa chromogenic assay. We used the chromogenic STA Liquid anti-Xa method and the measurements were performed on a STA-R Evolution^®^ coagulometer (Diagnostica Stago, Asnières-sur-Seine, France) according to the manufacturer’s instructions.^32^

### Statistical Analysis

Data were expressed as mean value with a 95% confidence interval (CI). The one way ANOVA with the post hoc test by Tukey correcting for multiple testing was used to calculate the differences in effect between the doses aPCC. Statistical calculations were performed by using SPSS version 26 (SPSS Inc., Chicago, USA). Statistical significance was set to *P* < 0.05.

## Results

Blood from 18 adult volunteers was collected. The concentration of apixaban in the spiked samples was 179 (95% CI 175, 183) ng/mL and the concentration of rivaroxaban was 206 (95% CI 190, 221) ng/mL.

### Thromboelastometry

CT was significantly prolonged in blood spiked with FXa inhibitors compared with baseline (*P* < 0.0001). The parameters CFT and MCF were not affected by apixaban and rivaroxaban, nor by aPCC.

**Figure 1.**
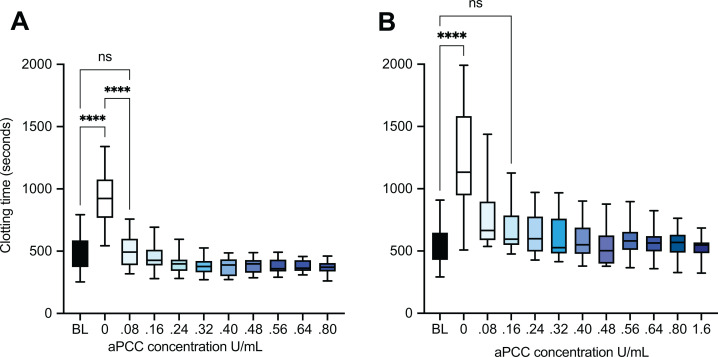
Thromboelastometry clotting time (CT) in whole blood spiked with apixaban (A) and rivaroxaban (B) before and after adding activated prothrombin complex concentrate (aPCC) in increasing concentrations. The lowest concentration of aPCC (0.08 U/mL) shortens CT to the level of baseline (BL) in apixaban-containing samples, and 0.16 U/mL shortens CT to baseline level in rivaroxaban-containing samples. Increasing the dose of aPCC does not give an additional effect. ****: *P* < 0.0001, ns: not significant.

CT was increased by adding apixaban from the value at baseline of 500 (95% CI 426, 573) seconds to 926 (95% CI 810, 1053) seconds. CT was shortened by successively adding aPCC. At the concentration of 0.08 U/mL, CT was reduced to 501 (95% CI 442, 561) seconds (*P* < 0.0001) matching the baseline level (*P* = 1.0) in the apixaban samples. Increasing the dose of aPCC did not cause further reduction of CT (*P* = 0.85). At the highest concentration 0.8 U/mL, which is corresponding to a dose of 50 U/kg, aPCC caused a shortening of CT to 368 (95% CI 334, 401) seconds which is 74% of baseline (Figure 1A).

Rivaroxaban increased clotting time from the baseline value of 548 (95% CI 468, 627) seconds to 1249 (95% CI 1036, 1463) seconds (*P* < 0.0001). aPCC caused a significant reduction of CT to 779 (95% CI 648, 910) seconds (*P* < 0.0001) at the concentration of 0.08 U/mL. aPCC at the concentration of 0.16 U/mL reduced CT to the level of baseline (*P* = 1.0). Increasing the concentration of aPCC did not give an additional effect on clotting time. Even by increasing the concentration by 20 times we did not obtain a significantly greater reduction of clotting time (*P* = 0.9) (Figure 1B).

### Thrombin Generation

**Figure 2.**
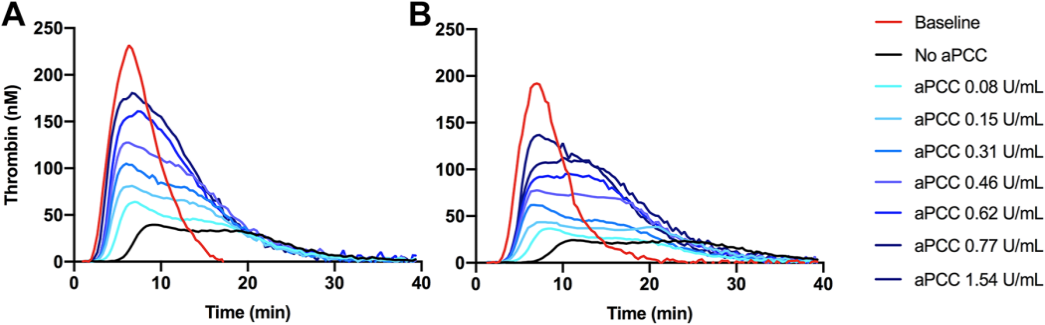
Thrombin generation curves after spiking apixaban (A) and rivaroxaban (B) containing samples with activated prothrombin complex concentrate (aPCC) in different concentrations. The curves are adjusted according to mean lag time, time to peak, peak thrombin generation, and endogenous thrombin generation (ETP). The red curves represent baseline, which is plasma not containing FXa inhibitor or aPCC. The black curves represent plasma spiked with apixaban (A) and rivaroxaban (B) with no aPCC added. The curves illustrate the concentration-dependent effect of aPCC in restoring thrombin generation in FXa inhibitor-containing plasma.

**Figure 3.**
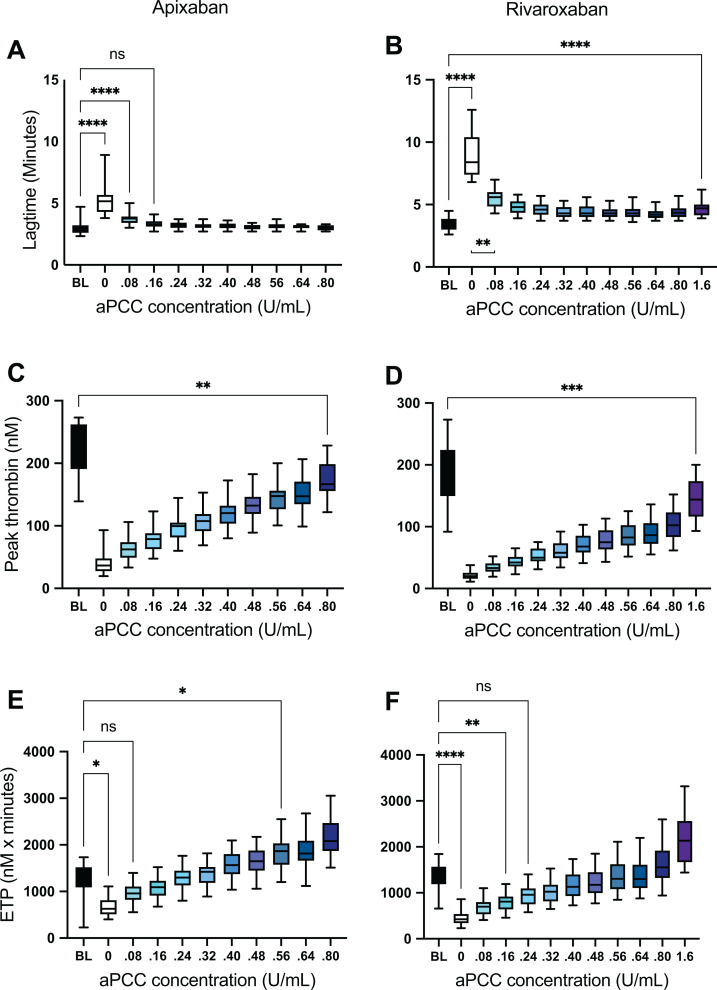
Thrombin generation parameters in apixaban- (A, C, E) and rivaroxaban- (B, D, F) spiked samples after addition of activated prothrombin complex concentrate (aPCC) in increasing concentrations. ****: *P* < 0.0001, ***: *P* < 0.001, **: *P* < 0.01, *: *P* < 0.05, ns: not significant.

Apixaban and rivaroxaban caused a reduction in thrombin generation (Figures 2 and 3). Lagtime increased from 3.0 (95% CI 2.7, 3.7) to 5.5 (95% CI 4.7, 6.4) minutes after adding apixaban and from 3.2 (95% CI, 2.9-3.6) to 8.5 (95% CI 7.5, 9.4) minutes after adding rivaroxaban to the samples. Peak thrombin generation was reduced by apixaban and rivaroxaban from 218 (95% CI 196, 240) nM to 41 (95% CI 31, 50) nM (*P* < 0.0001) and from 183 (95% CI 156, 210) nM to 21 (95% CI 18, 25) nM (*P* < 0.0001), respectively. ETP was reduced by apixaban and rivaroxaban from 1307 (95% CI 1129, 1482) to 680 (95% CI 572, 788) (*P* < 0.0001) and from 1330 (95% CI 1177, 1483) to 443 (95% CI 357, 528) (*P* < 0.0001), respectively.

Spiking the apixaban samples with aPCC at a concentration of 0.16 U/mL reduced lag time significantly to 3.3 (95% CI 3.1, 3.5) (*P* < 0.0001) which is at the level of baseline (*P* = 1.0) and the higher concentrations did cause a not further shortening (Figures 2A and 3A). Lag time of the rivaroxaban samples was significantly reduced by aPCC at all concentrations, but did not reach the level of baseline (Figures 2B and 3B).

aPCC increased peak thrombin generation in a dose-dependent relationship in both apixaban-and rivaroxaban-containing samples, but even at the highest concentrations (corresponding to 50 U/kg and 100 U/kg) it did not restore peak to the baseline level (Figure 3C and D).

ETP, however, was fully restored by aPCC. At the concentration of 0.08 U/mL, aPCC caused an increase in ETP in the apixaban samples to 1089 (95% CI 979, 1198) nM x minutes (*P* < 0.0001) which is the level of the baseline value (*P* = 0.5) (Figure 3E). At concentrations of 0.56 U/mL and higher, aPCC increased ETP to levels significantly higher than baseline (*P* < 0.05). Equally, in the rivaroxaban samples, aPCC at concentrations of 0.24 U/mL and above increased ETP to the level of baseline values (*P* = 0.2) (Figure 3F), but even the highest concentrations of aPCC did not overshoot the level of baseline.

## Discussion

In the present study we have demonstrated that aPCC at low concentrations reverses the effect of apixaban and rivaroxan *in vitro*. An aPCC concentration of 0.08 U/mL for apixaban and 0.16 for rivaroxaban, corresponding to doses of 5 U/kg and 10 U/kg, respectively, were sufficient to reduce thromboelastometry CT to baseline values. The thrombin generation parameter ETP was restored to baseline by an aPCC concentration of 0.08 U/mL for apixaban and 0.24 U/mL for rivaroxaban, corresponding to doses of 5 and 15 U/kg, respectively. These results may indicate that doses lower than suggested in guidelines are effective in reversing the effect of FXa inhibitors.

There are *in vitro* studies that have investigated the efficacy of aPCC in different concentrations to reverse the effect of FXa inhibitors.^20,21,25,33^ In previous work, our group has demonstrated that a concentration corresponding to 25 U/kg was sufficient to reverse the effect of apixaban.^25^ Moreover, one case series showed effect of aPCC 25 U/kg both by providing hemostasis in a surgical setting and measured by global coagulation assays.^24^ However, to our knowledge, no systematical investigation aiming to find the lowest effective dose has been performed previously. Therefore, in the present study aPCC was titrated starting with very low concentrations (corresponding to 5 U/kg) up to concentrations corresponding to higher doses than suggested in guidelines.

We chose a higher concentration of rivaroxaban than that of apixaban in the experiments in order to reflect clinical dosing where rivaroxaban is administered once daily leading to higher peak concentrations. This may explain the discrepancy in aPCC concentrations required to reverse the apixaban and rivaroxaban effects. Another difference in the measurement conditions was that rivaroxaban was dissolved in DMSO. Apixaban was soluble in water. One may question whether this small amount of DMSO may have affected the results. However, other studies have not demonstrated an effect of DMSO on coagulation assays.^34^

A limitation of the study is that the reversal effect of lower doses of aPCC than 0.08 U/mL has not been investigated. In apixaban-containing samples ETP and CT was brought back to baseline levels with aPCC in the lowest concentration which was 0.08 IU/mL, corresponding to a dose of 5 IU/kg. Thus, we do not know if even lower doses would have been sufficient to reverse the effect of apixaban. A study with titration of aPCC in concentrations from 0.01-0.08 IU/mL would be interesting in future work.

Current guidelines do not provide recommendations, but merely suggestions of aPCC dosing in treatment of major bleeding or preparation before emergency surgery in FXa inhibitor-treated patients. The dosing suggestions are much higher than the doses found effective in reversing the FXa inhibitor effect in the present study.^10–13^ The findings in the present study are of importance because lower doses aPCC might reduce the thrombotic risk associated with an overcorrection of the anticoagulant effect of FXa inhibitors. However, whether our results can be directly translated into a clinical setting has not been investigated, and clinical trials testing the use of aPCC in bleeding patients are warranted. So far, only smaller studies have tested aPCC in this setting, and different doses of aPCC have not been investigated.^17,18^ However, titrating aPCC dosage in a clinical setting does not seem feasible, as this would require an insurmountable number of inclusions. The *in vitro* findings of the present study may therefore help to determine aPCC dosage in future clinical trials.

## Conclusion

This *in vitro* study has shown that aPCC at a concentration of 0.08 U/mL and 0.24 U/mL reverses the effect of apixaban and rivaroxaban in peak concentrations, respectively. Whether this is the case *in vivo* remains unknown, but observational studies using higher doses of aPCC support this. Our results indicate that aPCC in doses no higher that 5-15 U/kg may be appropriate when reversing the effect of FXa inhibitors.
